# Excretion of urinary histamine and N-tele methylhistamine in patients with gastrointestinal food allergy compared to non-allergic controls during an unrestricted diet and a hypoallergenic diet

**DOI:** 10.1186/s12876-015-0268-4

**Published:** 2015-04-01

**Authors:** Martin Raithel, Alexander Hagel, Heinz Albrecht, Yurdaguel Zopf, Andreas Naegel, Hanns-Wolf Baenkler, Fred Buchwald, Hans-Wolfgang Schultis, Juergen Kressel, Eckhart Georg Hahn, Peter Konturek

**Affiliations:** 1 Department of Medicine 1, Functional Tissue Diagnostics, Gastroenterology, University Hospital Erlangen, University Erlangen-Nürnberg, Ulmenweg 18, Erlangen, 91054 Germany; 2 Medical Laboratory, Weiden, 92637 Germany; 3 Thuringia Clinics, Gastroenterology, Saalfeld, 07318 Germany; 4 Department of Medicine 3, Allergology, University Hospital Erlangen, University Erlangen-Nürnberg, Erlangen, 91054 Germany

**Keywords:** Gastrointestinally food allergy, IgE, N-methylhistamine, Urine histamine and methylhistamine excretion

## Abstract

**Background:**

Patients with gastrointestinal food allergy are characterised by increased production of mast cell derived mediators upon allergen contact and present often with unspecific symptoms. The aim of this study was to evaluate urinary histamine and methylhistamine excretion in patients with food allergy and to compare their values with food-tolerant controls.

**Methods:**

In a retrospective case control study the urinary excretion parameters were analysed from 56 patients (40.9, 19 – 58 years) in whom later food challenge tests confirmed food allergy. During their diagnostic work-up urine was collected during a 12-h period under an unrestricted diet with staple foods and a hypoallergenic potato-rice-diet (each 2 days). Healthy controls underwent the same diet types to define normal excretion parameters. Urinary histamine and n-methylhistamine were determined by ELISA or tandem mass spectrometry, respectively, and were expressed as median (25 – 75% range, μg/mmol creatinine x m^2^BSA).

**Results:**

During unrestricted diet urinary histamine was significantly higher in gastrointestinal food allergy than healthy controls (1.42, 0.9 – 2.7 vs 0.87, 0.4 – 1.3; p < 0.0001), while the difference between both groups became marginal during potato-rice diet (1.30, 0.7 – 2.1 vs 1.05, 0.5 – 1.5; p = 0.02).

N-methylhistamine was found to be significantly elevated in gastrointestinal food allergy both during unrestricted diet (7.1, 5.0 – 11.2) and potato-rice diet (5.7, 3.7 – 8.7) compared to controls (p < 0.0001). Interestingly, urinary methylhistamine excretion (p < 0.004) and clinical symptom score (p < 0.02) fell significantly when the diet was switched from unrestricted to hypoallergenic food, but was not correlated with symptom scores.

**Conclusions:**

In gastrointestinal food allergy significantly higher levels of urine histamine and methylhistamine excretion were found under unrestricted diet, reflecting an increased secretion of histamine due to offending foods. Measurement of urinary n-methylhistamine levels may help to find out patients with increased histamine production and/or food-allergen induced clinical symptoms, respectively.

## Background

Gastrointestinal complaints after the ingestion of certain foodstuff can occur in a large variety and can range as simple symptoms from bloating to severe symptoms like generalized skin reactions, gastroenteritis, colitis etc up to complications like bronchospasm and anaphylaxis. For differential diagnosis various clinical conditions have to be regarded and specifically examined such as food hypersensitivity, enzyme deficiencies, irritable bowel syndrome (IBS), Inflammatory Bowel Disease, dyspepsia, eosinophilic gastroenteritis and several others. However, objectification of immunologically mediated food hypersensitivity at the gastrointestinal level remains problematic, since atopy status is not a consistent feature in gastrointestinally mediated allergy (GMA), skin tests and allergen specific serum IgE detection may fail to show clear signs of food-specific sensitisation [1-5] and do not necessarily indicate symptomatic food allergy. In addition, allergic reactions of the gastrointestinal tract may follow either local intestinal IgE- or non-IgE mediated mechanisms or may occur from systemic IgE positive food allergy. Thus, several other functional tests using blood cells (basophil histamine, or leukotriene release), lymphocyte transformation tests or measurement of mediators in blood or serum [6-8] have been proposed to diagnose food hypersensitivity [3-7]. But only oral food challenge tests, referred to as the ‘gold standard’ for food allergy diagnosis, confirms the diagnosis, albeit in adults not all gastrointestinal reactions have been found to be IgE mediated [1,4,5,7-9].

However, until patients with recurrent gastrointestinal complaints due to food hypersensitivity undergo double-blind, placebo-controlled food challenge tests (DBPCFC) a substantial number of other differential diagnoses is to be excluded and finally, only a minority of patients underwent standardized food challenge procedures [1,3,8]. Since DBPCFC is both time and cost consuming, may put the patient at a more or less severe risk, allows only one food to be tested per 1 – 3 days [7,8,10], appropriate selection of individuals who should undergo challenge tests would be advisable. Since GMA includes heterogeneous patient subpopulations urinary histamine (UH) and urinary methylhistamine (UMH) excretion, as possible signs of manifest allergic disease, were evaluated in conjunction with clinical symptoms in a cohort of 56 patients with later confirmed GMA during two days of unrestricted diet (normal diet containing the usual staple foods) and subsequent two days of hypoallergenic potato-rice diet (elimination diet). The aim of this study was to define normal excretion rates of UH and UMH in a healthy non-allergic control group with food tolerance, to assess the rates of UH and UMH in food allergic individuals and to test whether one of these parameters might be used as potential screening parameter for GMA.

## Methods

### Study design and patient recruitment

Among a large number of patients with food related symptoms extensive differential diagnostics was performed to separate patients with various organpathological diseases, infectious disease and celiac disease from those with functional adverse food reactions.

**Figure 1:**
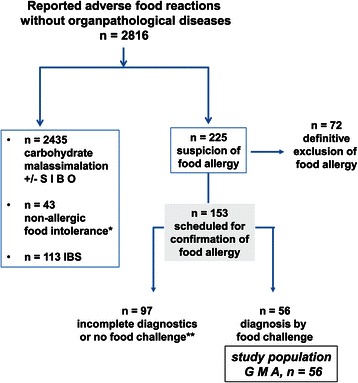
**A large number of patients with functional adverse food reactions were identified as carbohydrate malassimilation with/without small intestinal bowel overgrowth (SIBO), while a minority had non-allergic food intolerance or Irritable Bowel Syndrome (IBS).** Among the remaining 225 patients an allergic disease could be excluded definitively, while 153 had strong clinical suspicion of gastrointestinally mediated allergy (GMA) and were scheduled for confirmatory food challenge tests. However, only in 56 patients completed diagnostics was obtained and this group with confirmation of allergy is further described as study group GMA.

During the study period (2007 – 2011) 2816 individuals were identified with functional food reactions (Figure 1). Following diagnoses were confirmed in these patients, carbohydrate malassimilation, and/or small intestinal bacterial overgrowth, non-allergic intolerances and IBS or somatoforme diseases, respectively. Patients with prompt resolution of symptoms after diet or therapy were excluded from further allergy testing.

In 225 patients (7.9%) food allergy was initially suspected to induce adverse food reactions. Detailed clinical and diagnostic analysis (see below) did not show any signs of immunological sensitization or consistent evidence for food-induced symptoms in 72 patients.

The remaining 153 patients (5.4%) were further subjected to below listed diagnostic measures for confirmation of food allergy by oral food challenge tests, but complete data sets were only obtained from 56 patients in whom confirmation of GMA was later achieved by single- or double blind food challenge tests (BPCFCs). The main reasons for not completing the whole diagnostic pathway for confirmation of GMA in the 153 patients were (i) discontinuation before completing diagnostics (n = 14), (ii) non-compliance with the below listed functional diet test (n = 17), (iii) violation of the study protocol (n = 11; mostly alcohol consumption during the test days) or (iv) no willingness, no time or fear of oral food challenge tests (n = 111).

### Study group gastrointestinally mediated allergy (GMA)

Inclusion criteria for further confirmation of GMA were (i) food-related symptoms not attributable to another organic, functional or psychosomatic disease, (ii) persistent food-related symptoms despite adequate diet in carbohydrate malassimilation, (iii) history of atopy, previous typical signs of allergic disease (e.g. anaphylaxis, asthma bronchiale, urticaria, skin reactions, etc.), (iv) previous findings with pathological skin tests or elevated serum IgE and/or other immunological abnormalities (e.g. eosinophilia, mast cell infiltration etc.).

Exclusion criteria for investigating GMA and further involvement in the mediator excretion analysis were (i) another detectable organic disease (e.g. Inflammatory Bowel Disease), (ii) medication with immunosuppressants, mast cell regulating drugs, antihistamines or cromoglycate and biologicals (e.g. anti-IgE) or (iii) evidence of an underlying malignant disease, pregnancy, or chemoradiation.

**Figure 2:**
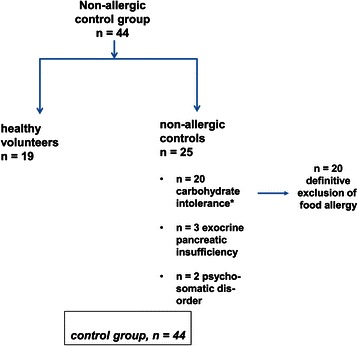
**The control group consisted of 19 healthy volunteers without any food related symptoms and 25 patients with non-allergic food intolerance.** As indicated for the 20 patients with carbohydrate malassimilation food allergy was specifically excluded by clinical diagnostics, skin tests, specific serum IgE and in unclear cases (n = 6) even with double-blinded, placebo-controlled food challenges with negative findings.

In 56 patients with the given inclusion and exclusion criteria full confirmation of GMA was achieved. This group is classified as GMA (study group) and will later be compared with a non-allergic control group (Figure 2) for the excretion of histamine and methylhistamine in urine.

The clinical characteristics, comorbidity, demographics and total serum IgE levels of the GMA group are listed in Table 1 and compared with the control group.

**Table 1:** **Demographics and clinical characteristics of the study groups with gastrointestinally mediated allergy (GMA) and the non-allergic control group**

	Gastrointestinally mediated allergy	Non-allergic control group*
	n = patients (%)	n = patients (%)
Number of patients	n = 56	n = 44
Age [years]	40.9 (19–58)	38.2 (16–76)
Sex [female/male]	35/21	20/24
Healthy volunteers	0/56	19/44
Carbohydrate malassimilation	29/56 (57.1)	20/44
Total serum IgE [kU/L]	74 (28–132)	49 (12–122)
Atopy	36/56	5/44
Confirmation of GMA by	56/56	0/56
DBPCFC/SBPCFC	41positive/15positive	6 DBPCFC negative/0
**Other comorbidities**		
Sensitization to grass/tree pollen	14/56	5/44
Associated gastroenterological diagnoses (esophagitis, ulcer, pancreatitis, colitis)	11/56	3/44
Oral allergy syndrome	5/56	0/44
Atopic dermatitis	5/56	0/44
Allergic rhinitis and/or conjunctivitis	4/56	7/44
Eosinophilic esophagitis	2/56	0/44
Anaphylaxis	2/56	0/44
Asthma bronchiale	1/56	0/44

*The control group consisted of 19 healthy volunteers without any food intolerance and 25 patients with non-allergic carbohydrate malassimilation (n = 20), exocrine pancreatic insufficiency (n = 3) and psychosomatic disease (n = 2). The group with gastrointestinally mediated allergy (GMA) includes 56 patients, in whom the allergic disease was proven oral food challenge tests. The different types of allergy found in this group are further differentiated and illustrated in Table 2.

Atopy status was defined as positive, when history or clinical manifestation of the patient gave evidence for seborrheic dermatitis, atopic dermatitis or eczema, asthma bronchiale and/or allergic rhino - conjunctivitis.

N = patient number, DBPCFC/SBPCFC double-blinded- or single-blinded, placebo-controlled food challenge test

### Clinical and allergological standard diagnostics in patients with gastrointestinally mediated allergy (GMA)

Following tests were done in each GMA patient (100%) with suspected food allergy to clearly exclude other diseases like infections, celiac disease, colitis, Inflammatory Bowel Disease, lymphoma, mastocytosis, and many others: Blood count, serology including transglutaminase antibodies, serum eosinophilic cationic protein, IgG, IgA, IgM, IgE, stool cultures, H2 breath tests, transabdominal sonography, upper and lower endoscopy and histology. Additional tests were done in 20 of 56 patients (35.7%) with determination of faecal elastase 1, in 8 patients (14.2%) with small bowel capsule endoscopy to rule out rare small bowel diseases [10], in 8 patients (14.2%) with measurement of plasma TNF and immune complexes to identify non-IgE mediated allergy types and in 4 patients (7.1%) with bone marrow biopsy.

Every patient was assessed on grounds of their history and detailed skin prick tests of environmental allergens (moulds, fibres, bacteria, pollen, dust) and staple food allergens (beef, egg, fish, fruit, pork, wheat, rye, soy, bran, milk, cheese and nuts). According to patients’ history some skin tests were extended for barley, coffee, oats, maize, peach and vegetables. Total and antigen-specific serum IgE detections were performed for staple foods and the putative allergens according to the patients’ history or skin tests. Case history, skin test reactions and serum antigen-specific IgE testing were supplemented with determination of intestinal antigen-specific IgE during endoscopically guided segmental gut lavage as described previously [11] before performing BPCFC. In the case of uncertainties about non-tolerated foods, tests were conducted only for staple foods. Patients with pathological H2 breath tests who developed further food-induced reactions despite strict carbohydrate avoidance, were also consequently suspected of having allergic or non-allergic hypersensitivity. Therefore, they were further subjected to above listed allergy diagnostics and later classified as GMA with associated carbohydrate malassimilation when immune sensitization signs and BPCFCs were positive.

Final confirmation of the diagnosis GMA was obtained by performance of the gold standard for food allergy diagnostics (given below) [1-4,12]. 15 single- (SBPCFC) and 41 DBPCFCs were undertaken in these patients to secure the diagnosis.

Final confirmation of the diagnosis GMA was obtained by performance of the gold standard for food allergy diagnostics (given below) [1-4,12]. 15 single- (SBPCFC) and 41 DBPCFCs were undertaken in these patients to secure the diagnosis.

Their clinical symptoms, signs of atopy, type of allergic reactions involved and the main causative food allergens detected during BPCFC are summarized in Tables 1 and 2 for the whole group. Table 3 shows all individual reactions of each allergy patient related to serum IgE, atopy status and the allergy type involved.

**Table 2:** **Allergy types according to Coombs and Gell, clinical reactions provoked by blinded, placebo-controlled food challenge tests (BPCFC) and causative allergens in the study group with gastrointestinally mediated allergy (GMA)**

Allergy type	Clinical reactions during food challenge	Eliciting allergens
	n = patients	n = patients
**Type I allergy**		
**(Systemic IgE)**		
25 patients	10 diarrhoea	7 nuts
18 atopics (72%) median serum IgE	4 pruritus, vomiting, abdominal pain, tachycardia	6 egg, wheat
		5 milk
		4 hazelnut, soy flour
		3 pollen associated fruits
		2 rye, celery, spice
		1 fish, maize, oat, barely, rice, peach, carrot, banana, pork
198 (84.5 – 405)		
	4 epigastric pain, bloating	
	2 nausea, hypotension, GI- bleeding	
	1 dyspepsia, flatulence, arthralgia, restlessness, fever, urticaria, abdominal colics,	
**Type I allergy**	12 diarrhoea	8 milk
**(Local IgE)**	10 abdominal pain	5 nuts, pork, egg, wheat
n = 22	5 bloating	3 rice, pollen associated fruits
11 atopics (50%)	4 colitis	2 soy flour, maize, celery
Median serum IgE	3 GI-bleeding	1 fish (salmon), beef, rye,
39 (23 – 77.5)	2 pruritus, eosinophilia, urticaria	hazelnut, moulds
	1 hypotension, dysphagia tachycardia, gastroesophageal reflux, ascites, edema	
**Type III allergy (serum immune complexes)**	3 diarrhoea	4 soy flour
	2 bloating, abdominal pain	2 pork
	1 GI-bleeding, tachycardia, hypotension, nausea	1 rye, rice
n = 4		
2 atopics (50%)		
median serum IgE		
38.5 (20 – 83)		
**Type IV allergy**	6 diarrhoea	5 rye
**(Cellular hypersensitivity)**	3 abdominal pain, pruritus	3 wheat, beef
n = 12	2 bloating, vomiting, flatulence, hypotension	2 milk, pork, soy flour, egg, soy bean
5 atopics (41.6%)		
Median serum IgE		
31 (10 – 57.5)		
	1 tachycardia, flush, GI-bleeding	1 nuts, chicken, maize, moulds, pollen-associated fruits

N = patient number, OAS oral allergy syndrome.

Atopy status was defined as positive, when history or clinical manifestation of the patient gave evidence for seborrheic dermatitis, atopic dermatitis or eczema, asthma bronchiale and/or allergic rhino - conjunctivitis.

For definition of the allergy type, the most dominant immunological signs were chosen to classify the ongoing allergic mechanisms in this population of patients with manifest gastrointestinally mediated allergy. However, some patients displayed symptoms that suggested more than one definitive type of allergy. Type I allergy (systemic IgE sensitization) was recognised when positive skin and/or antigen specific IgE levels were present in serum (>0.35 U/ml), type I allergy (local IgE sensitization) was diagnosed when intestinal lavage fluid contained elevated food antigen-specific levels of IgE (>0.35 U/mg protein) [11,23].

Type III allergy was found in 4 patients who showed formation of either IgA, IgM and/or IgE immune complexes during or after allergen application by blinded food challenge, while pre-challenge serum immune complexes were normal during potato-rice diet.

Type IV allergy was diagnosed or suspected in 7 patients and 5 with mixed allergy types who showed markedly increased production of serum TNF levels during or after food challenge, while pre-challenge TNF levels were normal during potato-rice diet. Additionally, in one patient type IV allergy was considered because of a positive antigen-specific lymphocyte proliferation test corresponding to the results of BPCFC.

**Table 3:** **List of individual symptoms, causative allergens, serum IgE and identified allergy types in each patient of the study group with gastrointestinally mediated allergy (GMA)**

Pat. no.	Main symptoms & allergen(ssensitivity)	Atopy status & serum-IgE		Type of allergy
1	Diarrhoea, flush, pruritus	-	76	Type I (systemic IgE)
	Soy flour, egg			
2	Abdominal pain, loose stools	+	210	Type I (systemic IgE)
	Bloating			
	Wheat, nuts			
3	Vomiting, diarrhoea	+	112	Type I (systemic IgE)
	Egg, wheat, soy			
4	Abdominal pain, urticaria	-	21	Type I (local IgE)
	Fish (salmon)			
5	Diarrhoea, abdominal pain	+	65	Type I (systemic IgE)
	Dyspepsia, vomiting			
	Hazelnut, tree pollens			
6	Diarrhoea	-	89	Type I (systemic IgE)
	Wheat, maize, barley			
7	Vomiting, loose stools	+	112	Type I (systemic IgE)
	Right lower quadrant pain			
	Rice, peach			
8	Profuse watery diarrhoea	-	398	Type I (systemic IgE)
	Milk, soy, fish			
9	Diarrhoea, bloating, tachy-	+	34	Type III (immune com-
	Cardia			Plexes present) or
	Pork, beef, soy			Type IV (?)
10	Pruritus, Rhinitis, tachycardia	-	54	Type I (systemic IgE)
	Bloating, diarrhoea			
	Nuts, milk			
11	Colitis, diarrhoea, arthralgia	-	66	Type I (systemic IgE)
	Oral allergy syndrome, rhinitis			
	Celery, carrot, tree & grass pollen			
12	Bloody diarrhoea, hypotension,	-	6	Type I (local IgE)
	And/or abdominal pain, bloating			Type III (IMMUNE
	rice, soy			Complexes)
13	Fever, diarrhoea, hypotension	+	722	Type I (systemic IgE)
	Nuts			
14	Diarrhoea, vomiting, abdominal	-	6	Type IV (cellular hyper-
	Pain			Sensitivity
	cereals (rye, wheat)			
15	Bloating, diarrhoea, eosinophilia	+	130	Type I (systemic Ige)
	Milk, egg			
16	Atopic eczema, diarrhoea, colitis	+	76	Type I (local IgE)
	Abdominal pain			Type IV (cellular hyper-
	Nuts, tree pollen			Sensitivity ?)
17	Rhinitis, vomiting, diarrhoea	+	111	Type I (local IgE)
	Nuts, egg			
18	Diarrhoea, bloating	+	28	Type I (local IgE)
	Wheat, milk			
19	Eosinophilia, bloating, diarrhoea	+	289	Type I (systemic IgE)
	Egg			
20	Gastrointestinal bleeding, colitis	+	80	Type I (systemic IgE)
	Wheat, hazelnut			
21	Chronic diarrhea, bloating, pain	-	12	Type IV
	Pork, beef			
22	Weight loss, diarrhea, pain	-	43	Type III (immune
	soy			Complexes)
23	Loose stools, abdominal pain	+	78	Type I (local IgE)
	Rice, egg			
24	Diarrhea, anaphylaxis	+	312	Type I (systemic IgE)
	Milk, egg			
25	Weight loss, malabsorption	-	34	Type I (local IgE)
	Milk, pork, wheat			
26	Tachycardia, abdominal pain	-	7	Type I (local IgE)
	Pork			
27	Colitis, abdominal pain, pruritus	+	92	Type I (local IgE)
	nuts, apple, tree pollen			
28	Chronic diarrhea	-	8	Type IV
	Rye,			
29	Gastroesophageal reflux	+	28	Type I (local IgE)
	Eosinophilia, intermittent diarrhea			
	milk, beef, tree pollen			
30	Anaphylaxis, urticaria	+	467	Type I (systemic IgE)
	Abdominal colics			
	Egg, milk			
31	Diarrhoea, bloating	+	28	Type I (local IgE)
	Wheat, milk			
32	Microscopic colitis, flush	-	32	Type IV
	Pruritus			
	Maize, rye			
33	Chronic diarrhea, bloating	+	77	Type I (local IgE)
	Abdominal pain, rhinitis			
	Milk, house dust mite, grass pollen			
34	Epigastric and abdominal pain	+	420	Type I (systemic Ige)
	Tachycardia, rhinitis, asthma bronchiale			
	Nuts, wheat, rye, oat			
35	Microscopic enteritis and colitis	-	22	Type I (local IgE)
	Malabsorption, pruritus			
	Egg, wheat, pork			
36	Eosinophilic enteritis, ascites	-	71	Type I (local IgE)
	Abdominal pain, loose stools			
	Maize, rice, fruits			
37	Allergic rhinoconjunctivitis,	+	412	Type I (systemic IgE)
	Epigastric pain, pruritus			
	Pollen, spice, celery			
38	Chronic diarrhea, nausea	+	123	Type I (systemic IgE)
	Soy, pork, rye, pollen			and type III (immune complexes)
39	Eosinophilic esophagitis	+	24	Type I (local IgE) and
	Atopic dermatitis			Type IV
	Egg, milk, pollen			
40	Vomiting, flatulence,	-	8	Type IV
	Hypotonia			
	Wheat, rye, soy bean, milk			
41	Gastric & duodenal ulcer	-	91	Type I (local IgE)
	Gastrointestinal bleeding			
	Eosinophilia			
	Soy flour, nuts, celery			
42	Weight loss, malabsorption	+	217	Type I (systemic IgE)
	Allergic rhinoconjunctivitis			
	Apple, nuts, latex			
43	Intermittent diarrhea	-	370	Type I (systemic IgE)
	Hazelnut			
44	Tachycardia, restlessness	-	28	Type I (systemic IgE)
	Loose stools			
	Wheat, pollen			
45	Oral allergy syndrome,	+	198	Type I (systemic IgE)
	Epigastric pain			
	Nuts, fruits, pollen			
46	Watery diarrhea	-	36	Type I (local IgE)
	Egg, milk			
47	Chronic diarrhea, flatulence	-	30	Type IV
	soy bean, beef, chicken			
48	Urticaria, oral allergy syndrome	+	42	Type I (local IgE)
	Colitis			
	Nuts, pollen			
49	Atopic eczema, proctitis	+	732	Type I (systemic IgE)
	Spice, pollen, hazelnut			
50	Gastrointestinal bleeding	+	74	Type I (local IgE) and
	From enterocolitis			Type IV
	Rye			
51	Dysphagia, epigastric pain	-	141	Type I (local IgE)
	Oral allergy syndrome			
	Fruits, hazelnut, celery			
52	Chronic diarrhea, abdominal	-	65	Type I (local IgE)
	pain and edema			
	pork, maize, moulds			
53	Lymphocytic colitis	+	122	Type I (systemic IgE)
	Pruritus, hypotonia			and type IV
	Banana, fruits			
54	Nausea, vomiting, flatulence	+	780	Type I (systemic IgE)
	Nuts			
55	Oral allergy syndrome, chronic	-	96	Type I (local IgE)
	Diarrhea			
	Wheat, rye			
56	Pruritus, weight loss,	-	41	Type IV
	Malabsorption			
	Wheat, rye, soy flour			

Atopy status was defined as positive, when history or clinical manifestation of the patient gave evidence for seborrheic dermatitis, atopic dermatitis or eczema, asthma bronchiale and/or allergic rhino - conjunctivitis.

For definition of the allergy type, the most dominant immunological signs were chosen to classify the ongoing allergic mechanisms in this population of patients with manifest gastrointestinally mediated allergy. However, some patients displayed symptoms that suggested more than one definitive type of allergy (see for example patient No. 9, 12, 16 etc.): Type I allergy (systemic IgE) was recognised when positive skin and/or antigen specific IgE levels were present in serum, type I allergy (local IgE) was diagnosed when intestinal lavage fluid contained elevated food antigen-specific levels of IgE (>0.35 U/mg protein) [11,23].

Type III allergy was found in 4 patients (no. 9, 12, 22, 38) who showed formation of either IgA, IgM and/or IgE immune complexes during or after allergen application by blinded food challenge, while pre-challenge serum immune complexes were normal during potato-rice diet.

Type IV allergy was diagnosed or suspected in 8 patients who showed markedly increased production of serum TNF levels during or after food challenge, while pre-challenge TNF levels were normal during potato-rice diet. Additionally, in one patient (no. 14) type IV allergy was considered because of a positive antigen-specific lymphocyte proliferation test.

### Confirmation of the diagnosis gastrointestinally mediated allergy (GMA) by oral food challenge tests

Blinded placebo-controlled food challenge tests (BPCFC) were done after extensive exclusion of various other differential diagnoses as indicated above. At least two weeks in advance of BPCFC, any antiallergic, immunosuppressive or steroid treatment had been discontinued for all patients and patients underwent a hypoallergenic diet at least 3 days before food challenges. Food challenges were only performed when patients had a clear resolution of their symptoms under the hypoallergenic diet with symptom scores < 3 points (Table 4). GMA was finally confirmed in each patient by blinded, placebo-controlled food challenge tests (BPCFC) adding the putative allergen to a basic diet containing rice, potato, oligopeptides (Survimed OPD, Fresenius, Germany) and tea. Allergens were freshly prepared and given to the patients via a nasogastric tube as described previously [4,11,12]. SBPCFC- and DBPCFC were performed in a standardised fashion, while patients were hospitalised and symptoms have resolved during hypoallergenic diet (score < 3 points). Food antigen was administered in three different doses over one test day. Initially, a 1/20 dilution of the native allergen solution was slowly administered via a pump at the nasogastric tube (volume up to 100ml for 1 hour), followed by 1/10 of the dose (for 2 hours) up to a volume of 200ml and finally, a dose of the full strength native allergen solution was provided with a volume of 200ml with fluid allergens or 0.6g/kg body weight of solid, but homogenized allergens, respectively, for 3 hours [11,12]. One single food antigen was tested per day. Food challenges were stopped in the case of ongoing symptoms exceeding a standardised symptom score of greater 6 points (Table 4), while reaction scores between 3 – 6 points were judged equivocal requiring re-challenge at another randomised provocation day [11,12]. When significant or equivocal reactions occurred, masked oligo-peptide diet as placebo was given the next day. Placebo consisted of an oligopeptide-diet (protein source: hydrolysed soybean, Survimed OPD, Germany), which was also used for base-line nutrition (minimum: 1800 kcal/day), in conjunction with a potato-rice diet in order to prevent a catabolic state [11-13]. A single blind challenge was performed in 15 patients (26.7%, patients unaware of provocation protocol), while a double-blind challenge was carried out in 41 patients (73.2%, patients and physicians unaware of the provocation protocol) [8,11,12]. Blinding of the food antigens was managed by nutritionists, who were responsible for the preparation and addition of the allergens to usually tolerated foodstuff or to the oligopeptide solution, respectively [4,11,12].

**Table 4:** **Erlangen symptom score used to quantify unrestricted diet, hypoallergenic diet and reactions during blinded, placebo-controlled food challenges (BPCFC)**

**I. General symptoms**							
**1. Reduction of general condition**				**2. Core temperature**			
Mild			1	Subfebrile; 37,5 to 38,0°C			1
Moderate			2	Low fever; 38,0 to 39,0°C			2
Severe			3	High fever; >39,0°C			3
Intolerable			4				
**II. Organotropic symptoms**							
**1. Eyes**				**2. CNS**			
**- Itching/burning**		**- Conjunctiva**		**- Headache/vertigo**		**- Sensitivity**	
Mild	1	Erythema	1	Mild	1	Paresthesia	1
Moderate	2	Swelling unilateral	2	Severe	2	Prickling	1
Continuous	3	Swelling bilateral	3	Migraine	3	Heat (feeling)	1
**3. Cardiovascular system**							
**Decrease of blood pressure (systolic)**				**Increase of heart rate**			
>10 mm hg			3	>10/min			3
>20 mm hg			6	>20/min			6
>30 mm hg			10	>30/min			10
**4. Respiratory systems**							
**- Nasal congestion**				**- Prickling/itching (nose & pharynx)**			
Unilateral			1	Occasional			1
Bilateral			2	With rubbing			2
Mouth breathing			3	Continuous			3
**- Rhinorrhea**				**- Sneezing**			
Occasional			1	Occasional			1
Frequently			2	Frequently			2
Continuous			3	Continuous			3
**- Larynx**				**- Pulmonary function test**			
Hoarseness			2	FEV1 80–60% / PEF <75% (baseline)			2
Laryngeal edema			5	FEV1 < 60% / PEF < 50% (baseline)			5
**- Bronchial obstruction (auscultatory)**				**- Cough**			
Expiratory wheezing			2	Occasional			1
In- & expiratory wheezing			5	Frequently			2
Massive obstruction (silent lung)			10	Staccato cough			3
**5. Gastrointestinal tract**							
**- Buccal cavity**				**- Abdomianl pain/cramps**			
Erythema/thumbness			1	Mild			1
Swelling			2	Moderate			2
Aphts			3	Severe			3
**- Esophagus**				**- Diarrhea**			
Pain			1	Loose			Number of stools x 1
Dysphagia/pyrosis			2	Liquid			Number of stools x 2
Bolus (feeling)			3	Bloody			Number of stools x 3
**- General**							
Meteorism			1				
Nausea			1				
Vomiting			Episodes x 2				
**6. Locomotor apparatus**							
**- Arthralgia (including spinal column)**				**- Arthritis (with joint effusion)**			
1 joint			1	1 joint			1
2–4 joints			2	2–4 joints			2
>4 joints			3	>4 joints			3
**7. Skin**							
**- Pruritus**				**-Erythema**			
Occasional			1	Mild			2
>2 minutes			2	Moderate, <50% body surface			5
Excoriations			3	Generalized, >50% body surface			10
**- Urticaria/angioedema**							
<3 hives			2				
3–10 hives			5				
Generalized, generalized flush			10				
	**Result:**						
	<3 points			Negative result			
	3–5 points			Questionable result			
	>6 points			Positive result			

Individual reactions and symptoms during the test days with unrestricted diet, potato-rice diet and during the food challenge tests were documented each for a 24 hours period and calculated for the corresponding diet and day, respectively.

Food challenges were only performed when patients had a clear resolution of their symptoms under the hypoallergenic diet with symptom scores < 3 points. Symptom scores higher than 6 points were judged as positive reactions and compared with the score during potato-rice diet [11,12].

Physicians selected the type of food to be tested either on the basis of the patients’ history, skin prick tests and serum or intestinal antigen-specific IgE tests or from the list of given staple foods. During the provocation procedure, the patients were provided with a peripheral venous line, and all medical staff involved was trained for medical intervention in case of an anaphylactic reaction. For the definition of all food allergic reactions, a modified scoring system (Table 4) was applied [11,12]. Main symptoms of patients evoked by the food allergen challenges are listed in Table 2 for the whole group and in Table 3 for each patient individually.

During BPCFC, at least one reproduction of an allergen induced clinical reaction and one or two placebo challenges were included for every patient. In total, after several provocation periods 1 – 9 food allergens (median 4, 1 – 14) have been tested per patient by BCFC with a cumulative median of 12 test days (3 – 28 days).

### Definition of allergy types

Food hypersensitivity was diagnosed as IgE-mediated GMA only when food-specific immune events were detected through positive skin tests (mean wheal diameter equal to the histamine reaction or > 3 mm in diameter [1,9,11-13]), serum antigen-specific IgE > 0.35 KU/L or greater (level 1 on the specific IgE scale, Phadia Cap-system, Phadia, Uppsala, Sweden) or through proof of intestinal IgE > 0.35KU/mg protein by endoscopically guided segmental lavage in conjunction with a positive challenge score > 6 points [11-13].

Food hypersensitivity was diagnosed as non-IgE-mediated GMA when 1) a reproducible clinical adverse reaction to the food antigen(s) occurred after allergen application (up to 24–48 hours) without evidence of cutaneous, systemic or local IgE sensitization, but 2) food-specific immune phenomena could be demonstrated during or after provocation (e.g. eosinophilic cationic protein, tumor necrosis factor, immune complexes etc., Table 1 [1,3,7-9,12]) and 3) other intolerance mechanisms have been excluded (histamine intolerance, salicylate sensitivity etc.).

### Control group

The control group of 44 individuals consisted of 19 healthy food-tolerating volunteers (43.1%) and 25 patients (56.8%) with non-allergic carbohydrate malassimilation (n = 20), exocrine pancreatic insufficiency (n = 3) and psychosomatic disease (n = 2).

All controls were also immunologically tested by skin prick tests for environmental allergens (moulds, fibres, bacteria, pollen, dust) and staple food allergens (beef, egg, fish, fruit, pork, wheat, rye, soy, bran, milk, cheese and nuts).

While 7 of the 19 food-tolerating volunteers had a history for extraintestinal allergy (rhinitis due to pollen and/or house dust mite sensitization), these 19 patients had non-food related symptoms. Among the 25 patients adverse reactions to various foods, above mentioned differential diagnostic steps including detection of food-specific IgE against staple foods and endoscopic-histological examination of the upper and lower gastrointestinal tract revealed carbohydrate malassimilation in 20 patients (45.4% of control group; lactose, fructose, and/or sorbitol malabsorption with clinical remission to diet), exocrine pancreatic insufficiency in 3 patients and psychosomatic disorders in 2 patients, but no other inflammatory, neoplastic, allergic or immunological disorder. However, 6 of these 20 malabsorptive patients underwent DBPCFC due to suspected non-IgE-mediated allergy, but no clinical reactions were recorded. Thus, these individuals were also classified as non-allergic controls with symptomatic carbohydrate malassimilation or pancreatic insufficiency. The clinical characteristics, comorbidity, demographics, total serum IgE and sensitizations of the control group are listed in Table 1.

### Urinary mediator excretion test during unrestricted diet and hypoallergenic diet

To evaluate urinary mediator excretion of histamine and methylhistamine patients with GMA (study group) and all persons of the control group underwent the following functional diet test: All individuals ingested during two subsequent days an unrestricted diet (day 1 and 2), followed by two further days with potato-rice diet (day 3 and 4). This functional diet test was performed before BPCFC with standardised food antigens to identify patients with enhanced histamine production.

The unrestricted diet was explained twice to the patients and controls, and it included ingestion of all listed staple foods (as given for skin prick testing and food-specific IgE), at least once, either at day 1 and/or day 2. The staple foods encompassed all common food groups of German nutrition habits. Among these staple foods following qualitative groups were allowed along with sugar and salt: Beef, egg, fish, fruit, pork, wheat, rye, soy, bran, milk, cheese, nuts, barley, coffee, oats, maize, apple, peach, banana and vegetables, but quantitative amounts were not recorded for practical reasons. Extraordinary food allergens like kiwi, Asiatic spices, curry, sesame etc. were not allowed as well as alcoholic beverages. During all test days symptoms were recorded, documented in a score and thus, patients improving in symptoms during potato-rice diet could be identified. 12-hour urinary mediator excretion was measured from each test day from an overnight collection period 6.00 p.m. to 6.00 a.m. [12,13].

During unrestricted diet patients ate the given staple foods and even their suspected foodstuffs until 2.00 p.m. and are allowed to drink 1.5 – 2.0 l water per test day. No immunosuppressive drugs, antiallergic medication nor consumption of alcohol was allowed during the urine collection days. After 2.00 p.m. patients were not allowed to ingest any foods up to the next morning to avoid food-induced urinary histamine contamination, and to adequately monitor endogenously produced histamine amounts during the 12-hour overnight collection period [12-15]. In the case of suspected severe allergic symptoms (anaphylaxis, hypotension, asthma, gastrointestinal bleeding etc.) this functional urinary mediator test was conducted on the ward or intensive care unit, respectively, with corresponding circulation monitoring.

During hypoallergenic potato-rice diet patients were allowed to eat only potato and rice, with or without salt and/or sugar until 2 p.m. and to drink 1.5 – 2.0 l water per test day. After 2.00 p.m. patients were not allowed to further ingest potatoes or rice. Urine collection period was also from 6 p.m. to 6 a.m. In three cases of rice allergy, the hypoallergenic diet was reduced to intake of potato only.

Urine from test day 1 and 2 (normal diet, all foodstuffs) as well as from test day 3 and 4 (potato-rice diet) was each prepared with 1 N HCL after the first urine portion to avoid bacterial contamination and all samples were processed as previously described [12,15,16]. Only urine samples with pH < 3 were taken for further analysis.

### Histamine and methylhistamine measurement from urine

Urinary histamine (UH) was measured in 12-hour urine samples by an ELISA according to manufacturers’ instructions (IBL Immunobiological Laboratories, Hamburg, Germany; sensitivity histamine 1.3 ng/ml) [17].

Urinary methylhistamine (UMH) was measured by tandem mass spectrometry (Medical Laboratory Buchwald/Schultis, Weiden, Germany). For tandem mass spectrometry 1 ml urine samples were neutralised and checked for pH, and purified after addition of an internal standard (1 – methylhistamine D3, Dr, Ehrensdorfer GmbH, Augsburg, Germany) by ion exchange (cation exchange Amberlite CG50, Sigma, Munich, Germany). After separation from matrix constituents urine eluates were then quantitatively analysed by tandem mass spectrometry (Ionics with HSID interface, Ionics MSV, Almere, Netherlands) using multiple reaction monitoring mode with the ion pairs 126.1/109.2 and 129.1/112.2, respectively, as internals standards [18].

The sensitivity of tandem mass spectroscopy was 2.6 ng/ml, test linearity was usually achieved between 7.8 – 2000 ng/ml with recovery of 97.7%.

As the rates of mediator excretion may be influenced by renal function, weight and body size, values were related to creatinine excretion. Concentrations of UH and UMH were expressed as μg/mmol creatinine x m^2^ body surface area (BSA) [12,15].

### Statistics

Statistical analysis was done by GraphPadPrims Software, using descriptive statistics with median and 25 – 75% interquartile range. Mean + 1 SD of controls was used to calculate the normal range of mediator excretion and correlations were done by Spearmann coefficient. Significant differences were calculated by Wilcoxon matched pairs test when comparing both diets within one group. For comparisons between the groups GMA and controls the Mann–Whitney test (*U*-test, unpaired) was used.

### Ethics approval and funding source

All patients and controls gave their informed consent and the study protocol was approved by the local ethics committee of the University Erlangen (No. 2500) and supported, in part, by grants from Marohn foundation (Erlangen, Germany).

## Results

### Characteristics of mediator test analysis and individual mediator excretion

Coefficients of intra- and interassay variation of 211 urine samples for detection of methylhistamine by tandem mass spectroscopy were 3.2 and 4.1%, respectively. The determination of H by ELISA showed an intra- and interassay variation of 4.1 and 9.8% respectively.

Intraindividual variation of UH and UMH of controls during both days with unrestricted diet was 21.2 + 14% and 17.6 + 8.1%, respectively. In the GMA group, intraindividual UH excretion showed a markedly higher coefficient of variation (39.1 + 17.9) than UMH (19.7 + 11.0%) during unrestricted diet at day 1 and 2.

As urine samples with pH > 3 might indicate bacterial contamination, 3/112 samples in the GMA group (2.7%) obtained from potato-rice diet could not be used for further analysis. Urine samples of all other groups and diet types had normal pH values below 3 and were further analysed.

### Control group

**Figure 3:**
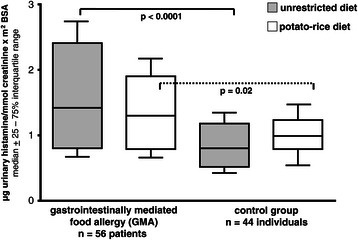
**Urinary histamine excretion (UH) in patients with gastrointestinally mediated allergy (GMA) and controls.** Horizontal lines represent the median of the group. Comparison of mediator excretion in both groups during unrestricted diet and a hypoallergenic elimination diet.

When testing for normal distribution of mediator excretion rates, only UH and UMH values of controls during unrestricted diet and UMH during potato-rice diet showed a Gaussian distribution, thus median and 25 – 75% interquartile range were given in the Figures 2 and 3 and non-parametric statistical tests were used.

**Figure 4:**
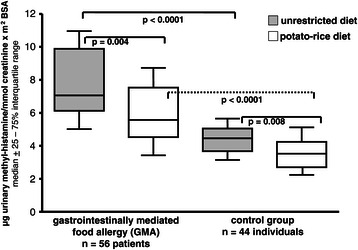
**Urinary methylhistamine excretion (UMH) in patients with gastrointestinally mediated allergy (GMA) and controls.** Horizontal lines represent the median of the group. Comparison of mediator excretion in both groups during unrestricted diet and a hypoallergenic elimination diet.

The median rates of UH and UMH excretion from controls are shown in Figures 3 and 4. In the control group there was no significant difference between both diet forms for UH, while UMH fell significantly during potato-rice diet in controls (p = 0.008).

**Figure 5:**
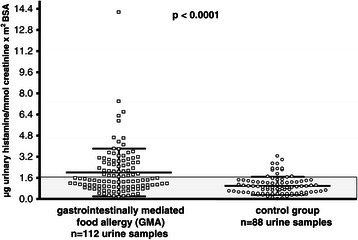
**Distribution of individual urine histamine (UH) values during unrestricted diet in 56 patients with gastrointestinally mediated allergy (GMA) and 44 controls.** The grey box represents normal values of UH (mean ± 1 SD of controls).

When analysing the distribution of individual UH excretion of controls during unrestricted diet (Figure 5, Tables 5 and 6), 13 of 88 urine samples (14.7%) showed UH levels greater than the mean + 1SD (0.99 + 0.68 = 1.67), while 75 urine samples (85.2%) were below this limit.

**Table 5:** **Sensitivity, specificity, positive and negative predictive values for the detection of urinary histamine (UH) or urinary N - methylhistamine (UMH) as non-invasive markers of gastrointestinally mediated allergy (GMA)**

Urinary histamine (UH) [μg/mmol creatinine x m^2^BSA]	Control group	Gastrointestinally mediated allergy (GMA)
>1.67*	13	50
≤1.67*	75	62
Total	88	112

*The significance level of 1.67 μg/mmol creatinine x m^2^ BSA was calculated from the mean + 1SD of controls during unrestricted diet. Using this criterion UH was found to reach following test characteristics:

UH sensitivity 50/112 = 44.6%; specificity 75/88 = 85.2%; positive predictive value 50/63 = 79.3%, negative predictive value 75/137 = 54.7%.

**Table 6:** **Sensitivity, specificity, positive and negative predictive values for the detection of urinary histamine (UH) or urinary N - methylhistamine (UMH) as non-invasive markers of gastrointestinally mediated allergy (GMA)**

Urinary methylhistamine (UMH) [μg/mmol creatinine x m^2^BSA]	Control group	Gastrointestinally mediated allergy (GMA)
>6.2*	12	70
≤6.2*	76	42
Total	88	112

*The significance level of 6.2 μg/mmol creatinine x m^2^ BSA was calculated from the mean. + 1SD of controls during unrestricted diet. Using this criterion UMH was found to reach following test characteristics:

UMH sensitivity 70/112 = 62.5%; specificity 76/88 = 86.4%; positive predictive value 70/82 = 85.4%, negative predictive value 76/118 = 64.4%.

**Figure 6:**
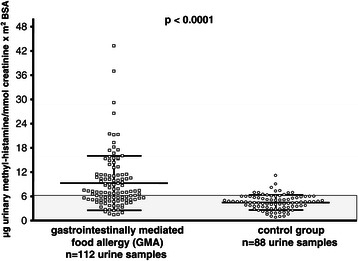
**Distribution of individual urine methylhistamine (UMH) values during unrestricted diet in 56 patients with gastrointestinally mediated allergy (GMA) and 44 controls.** The grey box represents normal values for UMH (mean ± 1 SD of controls).

When analysing the distribution of individual UMH excretion of controls during unrestricted diet (Figure 6, Tables 5 and 6), 12 of 88 urine samples (13.6%) showed UMH levels greater than the mean + 1 SD (4.4 + 1.8 = 6.2), while 76 urine samples (86.4%) were below this limit.

The median symptom score of control patients did not differ between unrestricted diet (1.8; 0 – 4.1) and potato-rice diet (2.1; 0 – 5.9).

### Gastrointestinally mediated allergy (GMA)

#### Clinical findings in GMA

All 56 proven food allergic individuals (100%) reported abdominal symptoms, nausea, pain, vomiting and/or diarrhoea (98%) after certain meals, while postprandial extraintestinal signs of allergy such as skin reactions, asthma bronchiale, pruritus and allergic rhinoconjunctivitis occurred only in a small percentage of patients (39%, Table 2).

Among the 56 allergy patients 29 individuals (57.1%) had a comorbid pathological H2 breath tests for either lactose, fructose and/or sorbitol (Table 1).

#### Urinary mediator results in GMA

The median rates of UH excretion are given in Figure 3 for the whole GMA group. There was no significant difference in UH between both diet forms in GMA patients.

But UH was each significantly elevated in GMA patients during unrestricted diet (p < 0.0001) and potato-rice diet (p = 0.02) compared to the corresponding diet in controls.

Among GMA patients 50 of 112 urine samples (44.6%) had UH levels of greater than the mean + 1SD of controls (>1.67), while 62 were below this limit. Sensitivity, specificity, positive and negative predictive value of UH are given in Tables 5 and 6.

The median rates of UMH excretion are given in Figure 4 for the whole GMA group, with significant differences among GMA patients ingesting an unrestricted diet versus a hypoallergenic elimination diet (p = 0.004). The differences in UMH between GMA and controls were highly significant when comparing both groups under unrestricted diet or hypoallergenic diet (each p < 0.0001).

Among GMA patients 70 of 112 urine samples (62.5%) had UMH levels of greater than the mean + 1 SD of controls (>6.2), while 42 were below this limit. Sensitivity, specificity, positive and negative predictive value of UMH are given in Tables 5 and 6.

The median symptom score of GMA patients showed a significant difference between unrestricted diet (4.4; 3 – 6) and potato-rice diet (3.0; 1 – 4, p = 0.03) as well as to controls for unrestricted diet (p = 0.02). However, there was no significant correlation between symptom score and UH or UMH excretion in GMA (r2 = 0.07, p = 0.6 and r2 = 0.08, p = 0.7, respectively).

#### Analysis of urinary mediator excretion rates according to IgE- and non-IgE induced types in GMA

When analysing all obtained individual UH and UMH values as shown in Figures 5 and 6 according to the presence of IgE- and non-IgE induced allergy types (Tables 2, 3 and 7) UH was each significantly elevated (p < 0.001) in the IgE- and non-IgE group compared to controls during unrestricted diet, but not during the hypoallergenic potato-rice diet (Table 7). There was no statistical significance between both diet types in the IgE- and non-IgE induced GMA groups for UH.

**Table 7:** **Urinary histamine (UH) and methylhistamine (UMH) excretion in relation to IgE- and non-IgE induced types of gastrointestinally mediated allergy (GMA) compared to the control group**

UH excretion	Unrestricted diet	Hypoallergenic potato-rice diet	Statistical significance vs control group unrestricted/hypoallergenic diet
IgE GMA	1.5 (0.9–2.6)	1.1 (0.8–2.2)	P < 0.0001/n.s.
Non-IgE GMA	1.4 (1.0–3.2)	1.46 (0.7–1.9)	P < 0.0001/n.s.
Control group	0.9 (0.4–1.3)	1.1 (0.6–1.6)	
**UMH excretion**			
IgE GMA	7.1 (5.9–11.2)	5.8 (3.9–9.7)	P < 0.0001/p < 0.0001
Non-IgE GMA	8.0 (4.8–11.3)	5.5 (3.1–8.2)	P = 0.003/p = 0.002
Control group	4.6 (3.2–5.8)	3.8 (2.3–5.1)	

UH and UMH excretion rates (median, 25–75th percentile; μg/mmol creatinine x m^2^ BSA) were separately analysed for the IgE- and non-IgE induced types of GMA.

Statistical significance between GMA groups and controls is given in the table.

In addition, there were no statistical significances for UH in the IgE- and non-IgE GMA group when comparing unrestricted diet versus hypoallergenic diet types (p = 0.09 and p = 0.27), while UMH in the IgE- and non-IgE GMA group revealed statistical significances (p = 0.04 and p = 0.002) between both diet types.

In contrast to UH, UMH was found to be uniformly and significantly increased in both allergy types (IgE- and non-IgE group) during the unrestricted diet and the potato-rice diet compared to controls (Table 7). Interestingly, UMH was also significantly different within the IgE- or non-IgE GMA group when switching from the unrestricted diet to the hypoallergenic diet (p = 0.04 and p = 0.002, respectively).

## Discussion

Gastrointestinal complaints after ingestion of various foodstuffs may result from a great spectrum of pathophysiological mechanisms, including infectious, toxic, immunological and non-immunological mechanisms as well as psychogenic reactions. However, at clinical presentation patients’ history and symptoms may be unspecific, atopy status may be inconsistent, sensitivity and specificity of antigen-specific IgE and skin tests vary in different patient subpopulations and the exact aetiology of food related symptoms often remains unclear [3,7-9]. Thus, the diagnosis of GMA requires a substantial degree of clinical suspicion, especially when patients present with recurrent postprandial abdominal symptoms and characteristic extraintestinal signs of allergic disease are not clearly observed. For this reason, the determination of biochemical mediator abnormalities resulting from degranulating allergic effector cells (e.g. basophils, mast cells) may facilitate the diagnosis of gastrointestinal food allergy. Thus, in an attempt to identify patients with histamine associated clinical symptoms, the results of a standardised functional urine mediator test were analysed in a cohort of patients in whom gastrointestinal food allergy has been confirmed by systemic and/or local immunological analysis as well as BPCFCs (Tables 1, 2, 3 and 4).

To imitate the clinical situation of patients with GMA, UH and UMH were collected first during a normal, unrestricted diet at two consecutive days (mostly at home, based on staple foods and putative allergens) which was suspected of inducing their complaints. Compared to controls who ingested also staple foods during unrestricted diet at day 1 and 2, patients with GMA exhibited significantly more symptoms than normal individuals under an unrestricted diet, and UH and UMH excretion were found to be highly significantly increased in allergic individuals. Although in the past, earlier attempts to diagnose GMA by histamine mediator excretion failed to show such clear results [14,19], these highly significant results may be the result of a strong standardization of this functional mediator test as described above (food ingestion up to 2 p.m., urine collection period from 6 p.m. to 6 a.m., each diet 2 days, fluid volume, water only etc. [4,13,15]) and an appropriate diagnostic work up for patient selection (Figure 1). Since the excretion of histamine, methylhistamine, imidazole acetic acid and its conjugates after food intake has been reported to occur within 1 – 3 hours after meal as a result from gastric acid stimulation in humans [20], intake of any foodstuffs within 4 hours before start of the urine collection period was not allowed in our functional mediator excretion study [4,12,13,15]. Before start of the urine collection period at 6 p.m. patients were advised to excrete their urine. Thus, urine samples collected after 6 p.m. and their mediator values obtained reflect more precisely the endogenous 12-hour histamine production overnight than exogenous histamine intake [13-15,20]. In addition, further standardisation was achieved by expression of all mediator excretion rates in relation to renal function and body surface area [12-15,21,22]. Finally, the results reported here arise from a cohort of fully confirmed GMA, since usual allergy tests were refined by search for intestinal IgE and proven by BPCFCs [4,7,9,11,12,16,21].

After two days of unrestricted diet clinical symptoms from GMA patients declined significantly, and UMH levels decreased in parallel and more pronounced than UH, indicating that the clinical effects of the hypoallergenic diet with potato-rice are accompanied at least, in part, by a lower rate of effector cell degranulation and/or histamine secretion. These findings could be found in patients with IgE-dominated as well as non-IgE dominated allergy types in adult GMA (Table 7), indicating that even in non-IgE GMA histamine release events and histamine production may be substantially involved [2,4-8,12,16,22]. But there was no correlation of UMH values with the clinical symptom score, indicating that several other mediators than histamine may also influence the symptoms of patients with gastrointestinal food allergy (e.g. prostaglandin D2, leukotrienes, platelet activating factor, tryptase etc. [5-7,12,16,22,23]).

While the elimination diet did not induce a clear decrease of UH excretion in allergic individuals and controls, UMH was found to decrease significantly and similarly in GMA patients (IgE- and non-IgE type) and controls during potato-rice diet, but in controls at a considerable lower UMH level. While GMA patients remained with their UMH levels mostly above 6.2 μg UMH/mmol creatinine x m2 BSA, controls were clearly below this differentiation level of 6.2 μg UMH/mmol creatinine x m2 BSA (Figure 3). The decline of UMH in controls may further indicate that the quantity of UMH excretion in humans reflects not only the rate of allergic effector cell degranulation and/or mast cell number or activity, respectively, but also several other intervening factors, like nutrient intake, protein content of the diet etc., resulting in a different extent of gastric acid stimulation by endogenous histamine dependent mechanisms [6,12-16,19].

However, UMH excretion was found to be highly significantly enhanced in both IgE- and non-IgE type of GMA patients than in controls under both diet forms, showed lower rates of intraindividual variation and it has been reported to reflect more precisely alterations of the histamine metabolism than histamine itself [12-14,21,22]. UMH presents as a possible indicator of systemic histamine production and it was found to increase in urine during early and late phase allergic asthmatic reactions [12,19,21-23]. The distribution of individual UMH levels in GMA and controls showed a considerable overlap, but in controls most patients (86.4%) were found to have UMH excretion rates lower than 6.2 (mean + 1SD). When using a UMH level of 6.2 as an additional diagnostic criterion to raise the suspicion of GMA or, at least, of an enhanced histamine production, respectively, 62.5% of patients showed UMH levels above this criterion, favouring further specialised allergy testing including an elimination trial and performance of BPCFCs, or, to consider other histamine related disease conditions like mastocytosis, myeloproliferative diseases etc. [4,6,16,20,21]. Thus, this standardised functional urine mediator test may help in future to directly identify patients with enhanced histamine and methylhistamine production and excretion during unrestricted diet, especially in patients with recurrent postprandial abdominal symptoms of yet unknown aetiology (e.g. chronic or intermittent diarrhoea, colic, pain etc., Table 1) both in patients with systemic signs of allergy (e.g. atopy, Table 1) and in conditions where local allergic mechanisms (e.g. entopy, Table 1) have previously been suggested (subpopulations of irritable bowel syndrome, microscopic colitis, persistent eosinophilia etc. [1,3,5,7,9,16,22-28]). However, increased excretion rates of UH and UMH are not specific for GMA, because several other diseases may be accompanied by persistently increased excretion of UMH like mastocytosis, active Inflammatory Bowel Disease, lymphoma, hematologic disorders etc. [6,14-16,19,25-28]. But compared to these diagnoses with persistently enhanced excretion of histamine and its metabolites, GMA patients were found to show a clear nutritive modulation of their UMH excretion within two consecutive days of elimination diet. Interestingly, such a dynamic, diet induced change of UMH excretion may serve in future as a substantial criterion to identify patients with GMA and to differentiate such patients from above mentioned disease conditions with persistently enhanced UMH excretion [14,16].

An important limitation of this study is the fact that the performance and detailed compliance of the unrestricted and hypoallergenic diet was not strictly supervised by online diaries and strict controls every day. Thus, dietary mistakes cannot be fully excluded and some allergy patients may have reduced the intake of a certain staple foods when perceiving symptoms or suspecting a food as allergic trigger. This may lead to a lower UMH production during unrestricted diet and may have influenced the diagnostic accuracy of UMH detection negatively, as we have shown clearly higher UMH levels during DBPCFC when freshly prepared allergens were applied solely [29]. But the aim of this functional mediator study was to explore UH and UMH excretion in patients with food-related symptoms during the typical clinical situation, mostly at home, when patients experienced their postprandial symptoms.

## Conclusion

Diagnosis of gastrointestinal food allergy is known to be difficult and needs a strict differential diagnostics and may be missed when serological, endoscopic and histological features are inconspicuous. Determination of UMH as an easy and non-invasive test was found to objectify whether histamine is involved in certain clinical symptoms. With a positive predictive value of 85.4% it may further indicate the gastroenterologist an underlying GMA. Patients with elevated excretion of UMH and gastroenterological symptoms should therefore further undergo detailed allergological testing at blood, skin and gut (local IgE) including food challenge procedures to avoid a rapid diagnosis of (idiopathic) irritable bowel disease, functional or psychosomatic disease [3,16,21,23,26,27].

## Abbreviations

BSA: Body surface area
GMA: Gastrointestinally mediated food allergy
UH: Urinary histamine
UMH: Urinary methylhistamine
DBPCFC: Double-blind, placebo-controlled food challenge tests
BPCFC: Blinded placebo-controlled food challenge tests
